# Piranha solution treatment: A facile method for improving the antithrombotic ability and regulating smooth muscle cell growth on blood contact materials

**DOI:** 10.3389/fbioe.2023.1166334

**Published:** 2023-03-13

**Authors:** Yuzhen Liao, Xiao Chen, Yutong Jiang, Chao Qu, Xiaoqi Liu, Ansha Zhao, Ping Yang, Nan Huang, Jiang Chen

**Affiliations:** ^1^ The Department of Ophthalmology, Sichuan Provincial People’s Hospital, University of Electronic Science and Technology of China, Chengdu, China; ^2^ Key Laboratory for Advanced Technologies of Materials, Ministry of Education, Institute of Biomaterials and Surface Engineering, Southwest Jiaotong University, Chengdu, China; ^3^ School of Stomatology of Guizhou Medical University, Guiyang, Guizhou, China; ^4^ Sichuan Provincial Key Laboratory for Human Disease Gene Study, The Department of Medical Genetics, The Institute of Laboratory Medicine, Sichuan Academy of Medical Sciences and Sichuan Provincial People’s Hospital, University of Electronic Science and Technology, Chengdu, China

**Keywords:** piranha solution treatment, free radical, anti-blood fouling, anticoagualant ability, smooth musce cell

## Abstract

Blood contact materials require strong anti-fouling capabilities to avoid thrombus formation. Recently, TiO_2_-based photocatalytic antithrombotic treatment has gained focus. Nevertheless, this method is restricted to titanium materials with photocatalytic abilities. This study offers an alternative solution that can be applied to a broader range of materials: piranha solution treatment. Our findings revealed that the free radicals generated by the treatment effectively altered the surface physicochemical properties of various inorganic materials, enhancing their surface hydrophilicity and oxidizing organic contaminants, thus improving their antithrombotic properties. Additionally, the treatment resulted in contrasting effects on the cellular affinity of SS and TiO_2_. While it significantly reduced the adhesion and proliferation of SMCs on SS surfaces, it significantly enhanced these on TiO_2_ surfaces. These observations suggested that the impact of the piranha solution treatment on the cellular affinity of biomaterials was closely tied to the intrinsic properties of the materials. Thus, materials suitable for piranha solution treatment could be selected based on the functional requirements of implantable medical devices. In conclusion, the broad applicability of piranha solution surface modification technology in both blood-contact and bone implant materials highlights its promising prospects.

## 1 Introduction

Blood contact materials require extremely high biofouling resistance to avoid triggering thrombosis on their surfaces when they come into contact with blood, leading to treatment failure (Kim et al., 2020; He et al., 2021). In recent years, the photo-induced antithrombotic properties of TiO_2_ due to photogenerated radicals had gained attention (Chen et al., 2014). UV-irradiated TiO_2_ could inhibit fibrinogen adhesion, thus inhibiting platelet adhesion and exhibiting excellent antithrombotic properties (Chen et al., 2015). However, this property was limited to titanium materials with photocatalytic properties. It would have been of significant application if similar antithrombotic properties could have been easily obtained on other materials.

The primary mechanism of the photo-induced antithrombotic properties of TiO2 could have been related to the generation of photogenerated free radicals, such as superoxide anion (O_2_^−^), atomic oxygen(O•), etc., which enhanced the hydrophilicity of the TiO2 surface and oxidized the surface organic adsorbates to form the organic residues containing carbonyl groups. (Liao et al., 2017). Inspired by this, in this study, we used a chemical reaction system, piranha solution, capable of rapid free radical generation (Cruz et al., 2022), for the free radical treatment of various common blood materials.

Piranha solution treatment was a classical method for cleaning material surfaces, based on the reaction of a mixture of concentrated sulfuric acid (97 wt%) and hydrogen peroxide (33 wt%) using a ratio of V_H2SO4_: V_H2O2_ = 3:1 (Ross et al., 1993). Through the complex process involving multiple reactions, many free radicals, mainly atomic oxygen (•O-), were produced in the piranha solution (Savaram et al., 2015). These free radicals oxidized and decomposed the material surface contaminants into water and CO_2_ through strong oxidation, leading to the cleaning purpose (Chen et al., 2011).

The following were some of the reactions that contributed to the generation of free radicals in Piranha solution: (Koh et al., 2012):

$$H_{2}{SO}_{4}+H_{2}O_{2}\to {\left[H3O\right]}^{+}+{HSO^{-}}_{4}+O•$$

In this study, we systematically evaluated the hydrophilicity, the charge of some common blood contact materials, and the surface chemical state after treatment with piranha solution. After that, we systematically evaluated the antithrombotic properties of these blood contact materials by platelet adhesion and activation assay and fibrinogen adhesion assay. Next, we investigated the *in vivo* antithrombotic properties of stainless steel treated with piranha solution in a rabbit *ex-vivo* circulation assay. Finally, we evaluated the affinity effect of TiO_2_ with SS on smooth muscle cells. The experimental results showed that piranha solution treatment rapidly improved the antithrombotic properties of various inorganic materials but was less effective on the organic material PTFE. The results of smooth muscle cells (SMCs) culture experiments showed that piranha solution-treated TiO_2_ indicated positive charge and promoted the adhesion and growth of SMCs. In contrast, piranha solution treatment did not affect SS’s charge, and piranha solution-treated SS significantly inhibited the adhesion and growth of SMCs. These results suggested that piranha solution treatment was a simple, universal way to improve the antithrombotic properties of blood contact materials. Also, piranha solution had different effects on the cytocompatibility of different materials. These results implied that different materials could have been selected and treated with piranha solutions to meet different medical implantable devices’ specific biological functional requirements. In summary, we believed the piranha solution had a broad application prospect as a surface modification tool.

## 2 Materials and methods

### 2.1 Preparation of the piranha solution-treated samplessolution-treated

**FIGURE 1:**
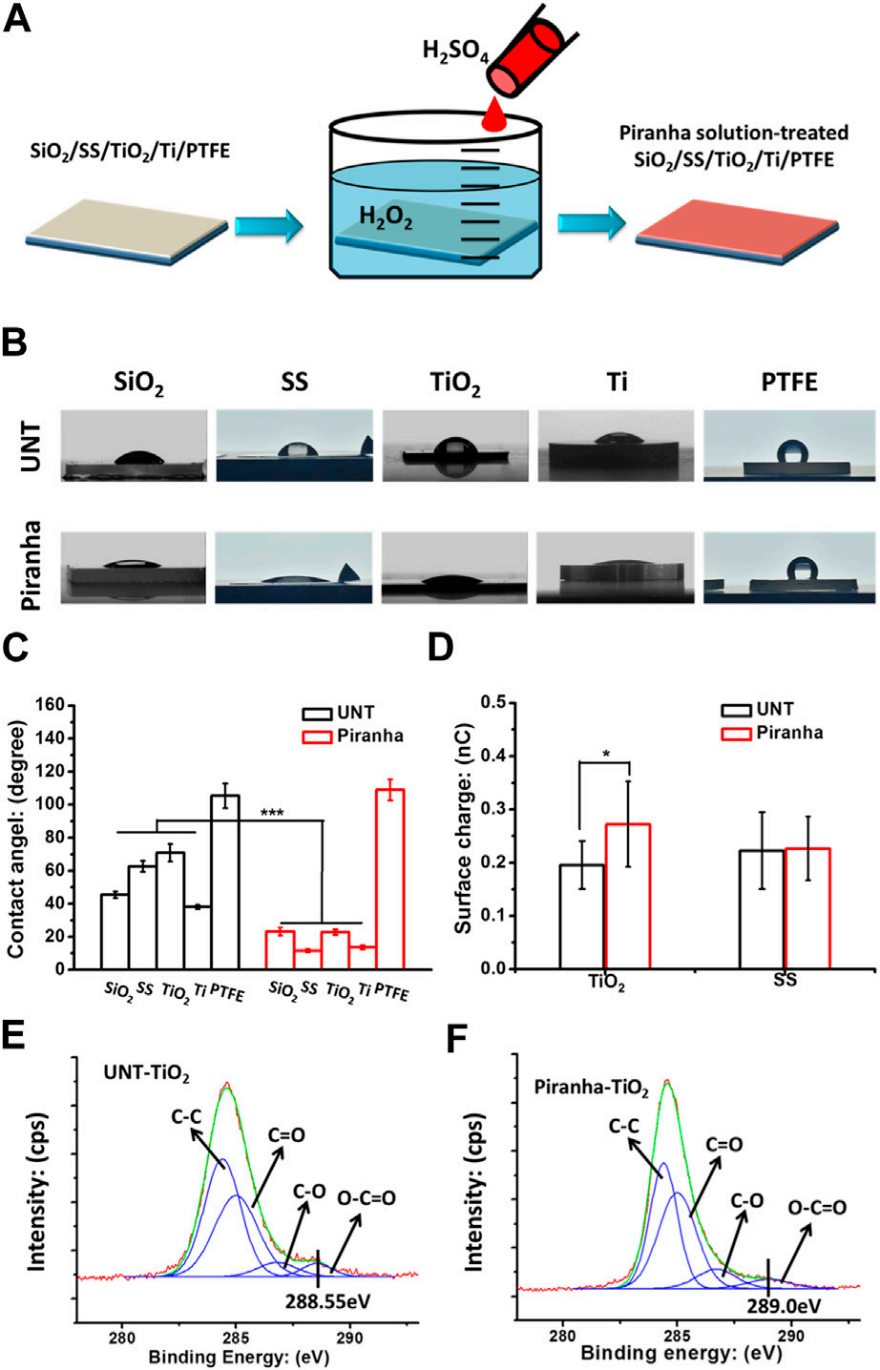
**(A)** Schematic diagram of the fabrication of the piranha solution-treated samples. **(B, C)** Water contact angle results. **(D)** Surface charges of samples. **(E)** XPS results, the C1s cloes view of the untreated TiO_2_(UNT-TiO_2_) and the **(F)** piranha solution-treated TiO_2_ (piranha-TiO_2_). (*p < 0.05).

Multiple types of biomaterials were treated with piranha solution in the following steps (Figure 1A).

(1) Put the samples into a glass container with one portion of H_2_O_2_ (30 wt%).
(2) Adding three portions of H_2_SO_4_ (98 wt%) to generate free radicals.
(3) The samples were removed, placed in RO water, and thoroughly cleaned in an ultrasonic cleaner three times to remove excess H_2_O_2_ and H_2_SO_4_.

### 2.2 Physicochemical characterization of samples

The hydrophilic behavior of each sample was evaluated before and after piranha solution treatment through the sessile drop method (5 μL droplet) with the drop shape analysis system (DSA 100, Kruss, Germany). The surface charge of TiO_2_ films and SS (18 × 18 mm^2^) before and after piranha solution treatment was measured using the EST111 Static Charge Meter (EST Electro-Static Test Co. Ltd., China). The surface chemical composition of the TiO_2_ films before and after piranha solution treatment was analyzed using X-ray photoelectron spectroscopy (XPS) on the XSAM800 (Kratos Ltd, UK). The instrument was powered by a monochromatic Al Kα X-ray source at 1486.6 eV with a voltage of 12 kV and current of 15 mA, operating at a pressure of 2 × 10^−10^ mB. The charge correction was carried out using the C 1s peak at 284.8 eV as a reference.

### 2.3 Platelet static adhesion test

The experiment utilized fresh whole blood from adult volunteers in good health, which was approved by the Medical Ethics Committee of the Affiliated Hospital of the University of Electronic Science and Technology & Sichuan Provincial People’s Hospital. The anticoagulant of choice was citric acid dextrose (ACD), and the blood-to-anticoagulant ratio was 1:9. The platelet-rich plasma (PRP) was obtained by centrifuging the blood at 1500 RPM for 15 min. Next, 50 μL of PRP was added to samples (7 × 7 mm^2^) that were placed in a 24-well plate. The samples were then incubated with the PRP for 1 h at 37°C. For removing non-adherent platelets, the samples were carefully rinsed three times with physiological saline. The adhered platelets on the samples were then fixed with 2.5% glutaraldehyde for 4 h. After undergoing gradient chemical drying, the number of adhered platelets on each sample was observed using optical microscopy (DM4000M, Leica, Germany) and quantified using ImageJ software with six random images (size = 500×) to obtain the platelet surface coverage. Lastly, the platelet’s morphology was examined using a scanning electron microscope (Quanta 200, FEI, Holland).

### 2.4 Platelet activation test

The GMP140 test was performed to evaluate the activation of adhered platelets. Samples of 7 × 7 mm^2^ were covered with 50 μL of fresh human platelet-rich plasma (PRP), collected from a healthy adult volunteer, and centrifuged at 1500 rpm for 15 min. The PRP was incubated at 37°C for 1 h. After the incubation, the samples were washed thoroughly with phosphate buffered solution (PBS) and then blocked with a 1 wt% bovine serum albumin (BSA) solution in PBS at 37°C for 30 min. Afterward, the samples were rewashed and covered with 20 μL of mouse anti-CD62p (1:100), also known as GMP-140 (MCA796GA, Serotec Co., Japan) incubated at 37°C for 1 h. After three times washing with PBS, the samples were incubated with 20 μL of horseradish peroxidase-conjugated sheep anti-mouse polyclonal antibody (HRP, Catalog No.: 074–1806, KPL Co., South Korea) solution for 60 min at 37°C. The samples were then washed thoroughly with PBS, and 70 μL of chromogenic substrate 3,3′,5,5′-tetramethylbenzidine (TMB, Bioss, China) solution (diluted 1:4 in PBS) was added to react with the sample surface. After 10 min, 50 μL of 1 M H_2_SO_4_ was used to stop the reaction, and the optical density at 450 nm was measured using a microplate reader. The relative amount of adsorbed fibrinogen was quantified based on the calibration curve (Mou et al., 2022).

### 2.5 Fibrinogen adsorption test

Firstly, 40 μL of fresh human platelet-poor plasma (PPP) was added to samples (7 × 7 mm) and incubated at 37°C for 1 h. The PPP extraction process is described in detail elsewhere (He et al., 2021). Then, the samples were rinsed thoroughly with PBS. Then, the samples were immersed into the block solution (1 wt% bovine serum albumin (BSA) in PBS) at 37°C for 30 min. Subsequently, the samples were thoroughly rewashed and covered with 20 μL of Horseradish Peroxidase (HRP)-labeled mouse antihuman fibrinogen monoclonal antibody (primary antibody, diluted 1:200 in PBS; Sigma, St. Louis, MO) and incubated at 37°C for 1 h. After thoroughly washing the samples, 70 μL of chromogenic substrate 3,3′,5,5′- tetramethylbenzidine (TMB) solution (diluted 1:4 in PBS) was added to the surfaces of the samples. 10 min later, 50 μL of 1M H_2_SO_4_ was added to stop the reaction, and a microplate reader was used to determine the optical density at 450 nm. The relative amount of adsorbed Fgn was quantified according to the calibration curve.

### 2.6 Evaluation of anti-thrombogenicity *ex-vivo*

The animal experiments in this study followed the regulations set forth by China’s laboratory animal management standards and the Medical Ethics Committee of the Affiliated Hospital of University of Electronic Science and Technology and Sichuan Provincial People’s Hospital. White New Zealand rabbits weighing between 4.0 and 4.5 kg were used. The SS foils, with or without piranha solution treatment, were rolled up and individually placed in the center of a polyvinyl chloride catheter. The foils were securely attached to the inner wall of the catheter. One end of the catheter with the sample was connected to the carotid artery, while the other end was connected to the jugular vein. Blood flowed through the catheter and over the sample surface for 45 min. Afterward, the catheter was disconnected and removed from the animal. It was then rinsed with 0.9 wt% saline, and the sample was collected and photographed. The samples were fixed in 2.5% glutaraldehyde solution for 12 h, underwent dehydration, and were weighed to calculate the weight of each thrombus. Finally, Scanning Electron Microscopy (SEM, Quanta 200; FEI, Holland) was used to observe the thrombus morphology on each sample surface (Yang et al., 2020).

### 2.7 Cultureing of smooth muscle cells (SMCs)

*In vitro* culture of smooth muscle cells (SMCs) was carried out using SMCs extracted from human umbilical cord veins. The sterilized TiO_2_ and SS samples with or without piranha solution treatment were placed in a 24-well cell culture plate, with each well containing 1 mL of medium with a concentration of 1.5 × 10^4^ cells/mL of SMCs. The samples were then incubated at 37°C under a 5% CO_2_ atmosphere for 1 day and 3 dyas. Following incubation, the samples were washed three times with PBS to eliminate unattached cells. Subsequently, the samples were fixed with 2.5% glutaraldehyde for 12 h. The SMCs were then stained with rhodamine and analyzed under a fluorescence microscope (IX51, Olympus, Japan). The cell surface coverage of the SMCs was studied by acquiring at least six random images per sample and analyzed using ImageJ and SPSS software.

### 2.8 Statistics

The experiments were replicated three times, yielding a total of three data points (*n* = 3) for each test. For determining the statistical significance between the sample groups, the data were analyzed using one-way ANOVA and LSD posthoc test with SPSS11.5. A significance level of *p* < 0.05 was established as the benchmark to determine statistical significance.

## 3 Results and discussion

### 3.1 Characterization of materials

As shown in Figure 1A, the study investigated the general applicability of the piranha solution treatment to various biomaterials, including silicon dioxide (SiO_2_), stainless steel (SS), titanium dioxide film (TiO_2_), titanium metal (Ti), and polytetrafluoroethylene (PTFE). The results in Figures 1B, C showed that after the piranha solution treatment, the water contact angle of the inorganic materials all significantly decreased, while the water contact angle of PTFE did not change significantly. The decrease in hydrophilicity of the inorganic materials could be due to the introduction of hydrophilic hydroxyl (-OH) groups on the material’s surface by the treatment. In contrast, the water contact angle of PTFE might be due to its highly conserved structure, which is difficult to be activated effectively with a piranha solution.

Figure 1C selected SS and TiO_2_ from the various materials as representatives for surface charge properties. SS was used as a representative of conventional inorganic materials, while TiO_2_ was used as a representative of inorganic materials with unique surface charging properties, such as Ti, ZrO, etc., which change their surface charge from negative to positive when cleaned (Hori et al., 2010; Iwasa et al., 2010). The results showed that the piranha solution treatment did not change the surface charge of SS, but significantly enhanced the charge-positive property of TiO_2_. This could be due to the cleaning effect of the piranha solution treatment on the TiO_2_ surface.

The results of the high-resolution spectrum of C1s on the TiO_2_ surface, shown in Figures 1D, E, indicated that the piranha solution treatment significantly oxidized the oxygenated hydrocarbon adsorbates on the TiO_2_ surface, as the shoulder peak at 288.55 eV on the TiO_2_ surface was red-shifted to 289.0 eV after the treatment (Cui et al., 2021). This phenomenon is consistent with that of TiO_2_ after UV irradiation.

### 3.2 Analysis of antithrombotic property

**FIGURE 2:**
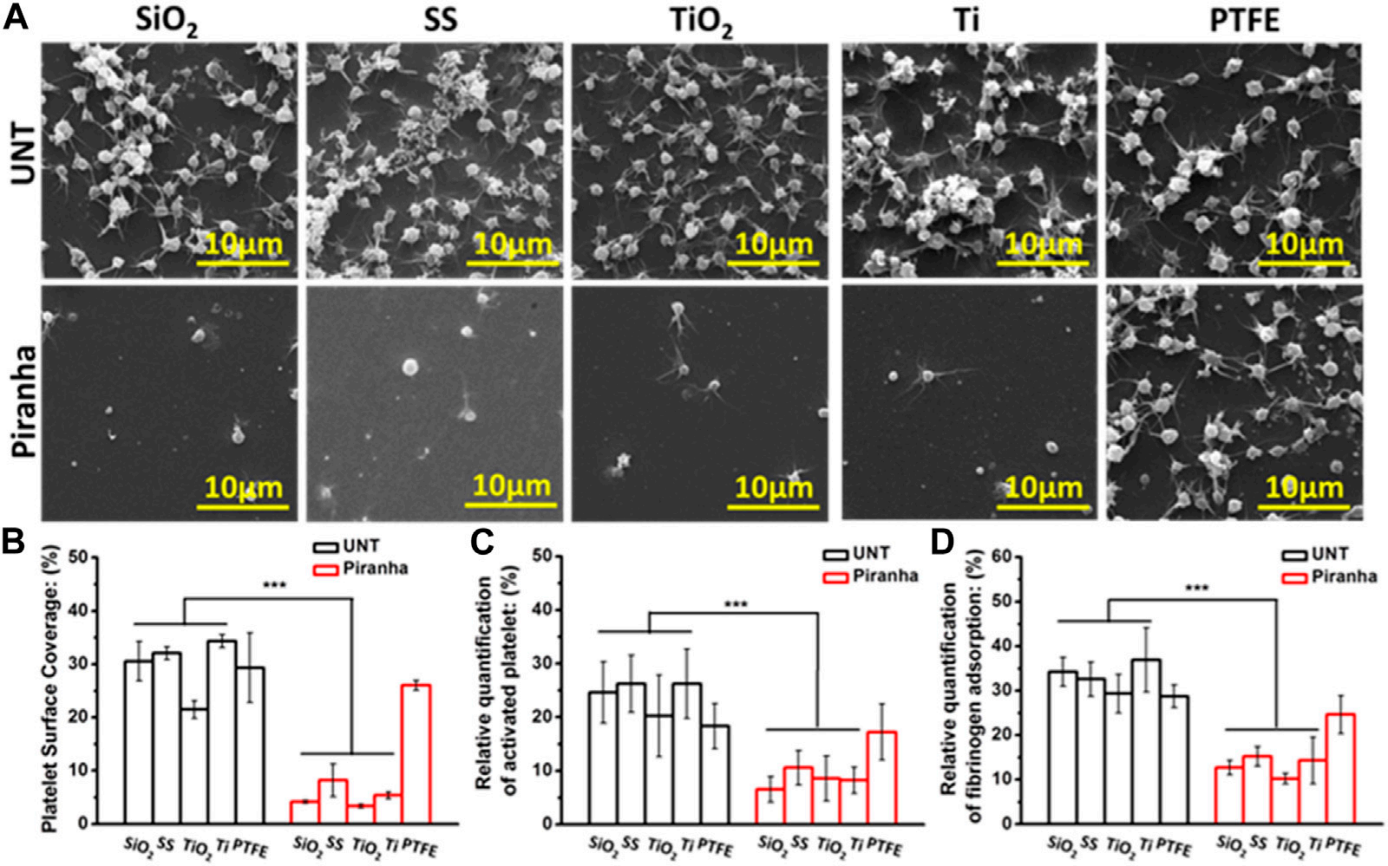
*In vitro* platelet adhesion assay. **(A)** SEM images **(B)** platelet adhesion area ratio (Sp), **(C)** platelet activation, **(D)** fibrinogen adsorption (**p < 0.01, ***p < 0.001).

As shown in Figure 2A, in the untreated (UNT) group, platelets heavily adhered to and were significantly activated on the surface of all types of materials, causing platelet spreading and agglomeration. In contrast, in the group treated with the piranha solution, the number of platelet adhesions on SiO_2_, SS, TiO_2_, and Ti surfaces was significantly decreased, the activation of platelets was effectively suppressed, and the platelet morphology was mainly characterized by protruding pseudopods. However, the treatment with the piranha solution did not significantly improve the platelet adhesion and activation behavior on PTFE. Combined with the results of the water contact angle (piranha solution treatment could not improve the hydrophilicity of PTFE), it can be speculated that piranha solution treatment did not significantly change the surface chemical state of PTFE, resulting in the inability to change the biological properties of PTFE.

The Platelet Surface Coverage (PSC) could reflect the number of platelet adhesion and the degree of activation in a comprehensive manner. As shown in Figure 2B, the PSC of the piranha-treated samples, except for PTFE, decreased significantly compared to the PSC of the untreated samples. The decrease rate of PSC of the same material treated with the piranha solution was defined as DPSC and calculated using the following equation:

$$D_{PSC}=\left({UNT}_{PSC}-{Piranha}_{PSC}\right)/{UNT}_{PSC}\times 100$$ (1)

Then, the results showed that SiO_2_-D_PSC_ = 86.3%, SS-D_PSC_ = 74.4%, TiO_2_-D_PSC_ = 84.3%, TiO_2_-D_PSC_ = 84.4%.

As shown in Figure 2C, the Platelet Activation Rate (PAR) had a similar pattern to the PSC, and the piranha solution treatment reduced the PAR of all materials except PTFE. The decrease rate of PAR of the same material after treatment with the piranha solution was defined as DPAR, and the results showed that SiO_2_-D_PAR_ = 73.53%, SS-D_PAR_ = 59.7%, TiO_2_-D_PAR_ = 57.8%, TiO_2_-D_PAR_ = 68.7%.

As the first coagulation factor, fibrinogen directly affects platelet adhesion and activation. As shown in Figure 2D, the piranha solution treatment significantly reduced the Fibrinogen Adsorption Rate (FAR) of SiO_2_, SS, TiO_2_, and Ti. The decrease ratio of FAR of the same material after treatment with the piranha solution was defined as D FAR, and the results showed that SiO_2_-D_FAR_ = 63.0%, SS-D_FAR_ = 53.2%, TiO_2_-D_FAR_ = 65.2%, TiO_2_-D_FAR_ = 61.3%.

In summary, the *in vitro* hemocompatibility results indicated that the piranha solution treatment could effectively enhance the antithrombotic properties of various inorganic materials and had a broad potential for application.

**FIGURE 3:**
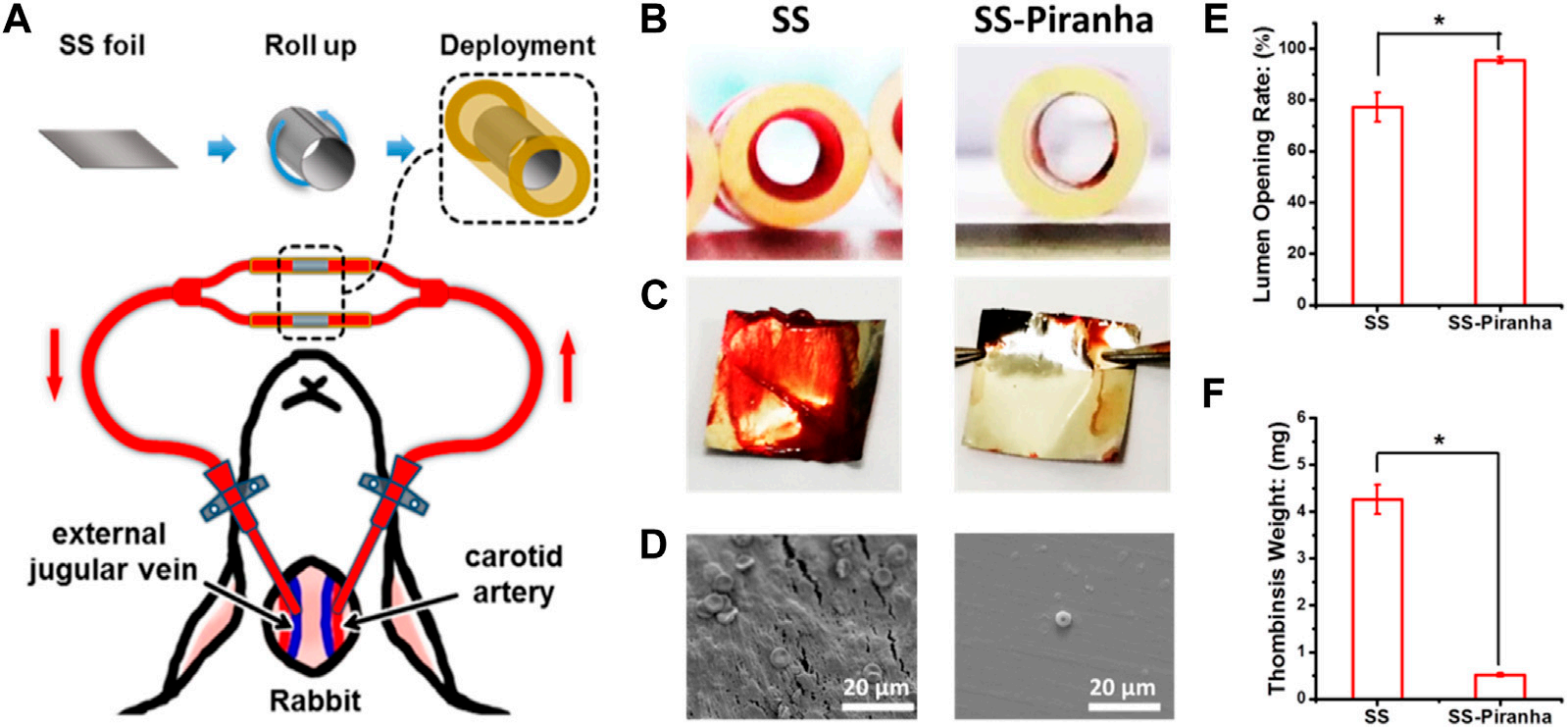
**(A)** Schematic diagram of the *ex-vivo* blood circulation experiment. **(B)** Photograph of the catheter and **(C)** SS foil after the experiment. **(D)** SEM image of the SS foil **(E)** Lumen opening rate, **(F)** the weight of the thrombus formed on the SS foil.

This study evaluated the *in vivo* anti-thrombogenic properties of SS treated with a piranha solution using a rabbit arteriovenous shunt model. As shown in Figure 3A, the samples were rolled up and tightly attached to the lumen wall of the circuit and the residual thrombus formation was measured after 45 min of cycling.

The results, as depicted in Figures 3B, C, showed that thrombin formed on the SS-UNT, inducing thrombotic occlusion of the circuit. As shown in Figure 3E, the lumen opening rate of the SS-UNT was lower than 80%, while that of the SS-piranha was about 92%. As shown in Figure 3F, the thrombosis weight of the SS-UNT was about 4.2 mg, while that of the SS-piranha was fewer than 0.5 mg.

These results indicate that the piranha solution treatment significantly enhanced the anti-thrombogenic properties of SS, which was widely used in blood-contacting devices such as the vascular stent and inferior vena cava filter.

### 3.3 Static ECs culture test

Smooth muscle cells (SMCs) were considered important vascular wall cells and key players in the physiological homeostasis of vascular tissue (Qiu et al., 2021). Studying the interaction between materials and SMCs had important implications for long-term vascular implant devices, such as vascular stents (Qiu et al., 2019). Because the overproliferation of SMCs was the leading cause of restenosis in the lumen of vascular stents, materials were generally desired to have the ability to inhibit SMC growth (Yang et al., 2018).

**FIGURE 4:**
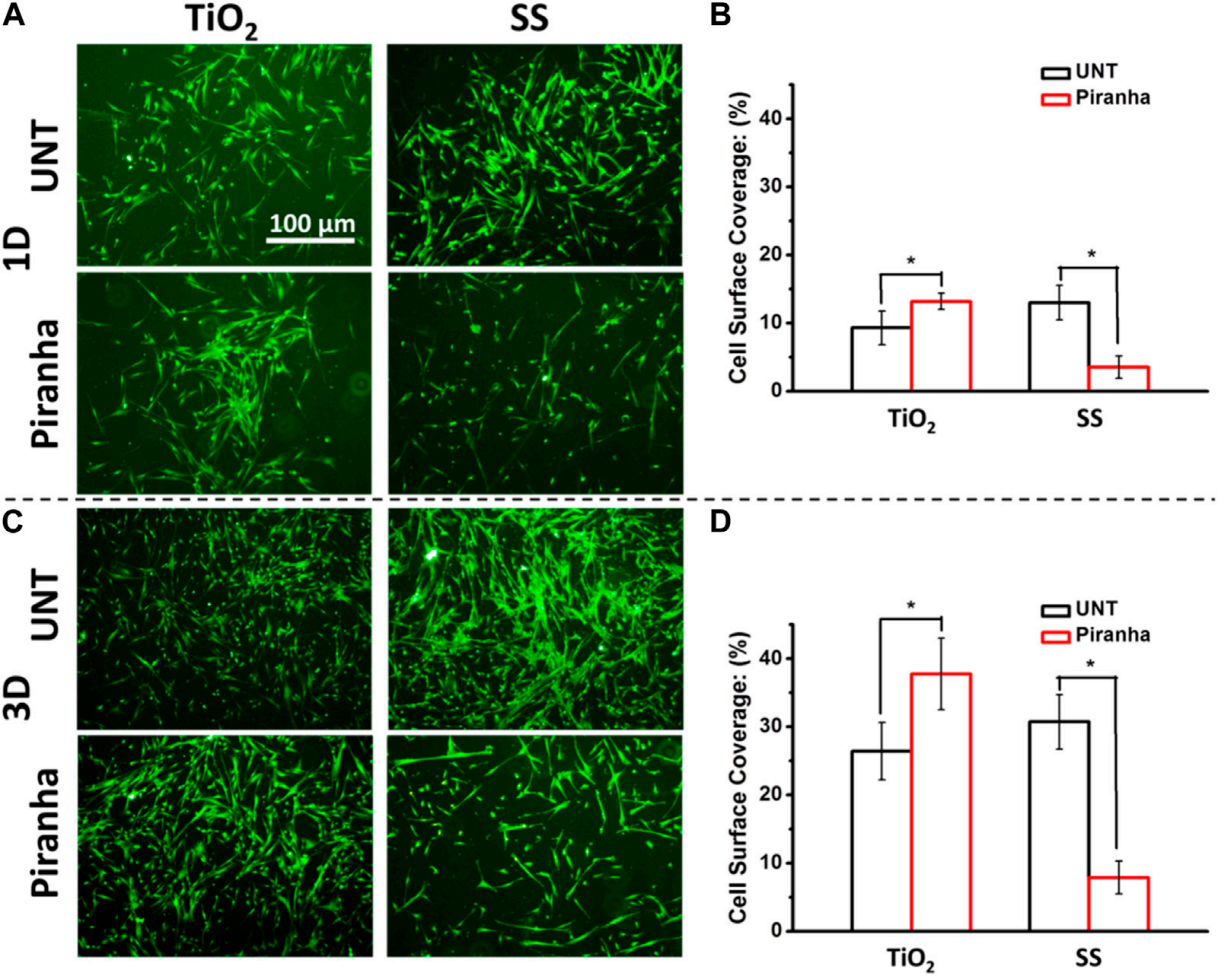
**(A)** Fluorescence photographs of adhered SMCs **(B)** SMCs surface coverage after being cultured for 1 day (I) SMCs surface coverage after being cultured for 3 days (*p < 0.05).

As shown in Figures 4A, B, after a day of incubation, the number of SMCs on SS-piranha was significantly less compared to that on SS-UNT. However, on TiO_2_-piranha, the number of SMCs was significantly higher compared to that on SS-UNT. Cell surface coverage (CSC) statistics showed that the CSCs of TiO_2_-UNT and TiO_2_-piranha were 9.3% and 13.2%, respectively, while the CSCs of SS-UNT and SS-piranha were 12.9% and 3.6%, respectively.

As shown in Figures 4C, D, after 3 days of incubation, the number of SMCs on SS-piranha was significantly less compared to that on SS-UNT. On the other hand, the number of SMCs on TiO_2_-piranha was significantly higher compared to that on SS-UNT. Statistics showed that the CSCs of TiO_2_-UNT and TiO_2_-piranha were 26.4% and 37.7%, respectively, while the CSCs of SS-UNT and SS-piranha were 30.7% and 7.9%, respectively.

These results indicated that the effect of the affinity of materials to SMCs after piranha solution treatment might have different or even diametrically opposite effects depending on the inherent properties of the materials themselves.

**FIGURE 5:**
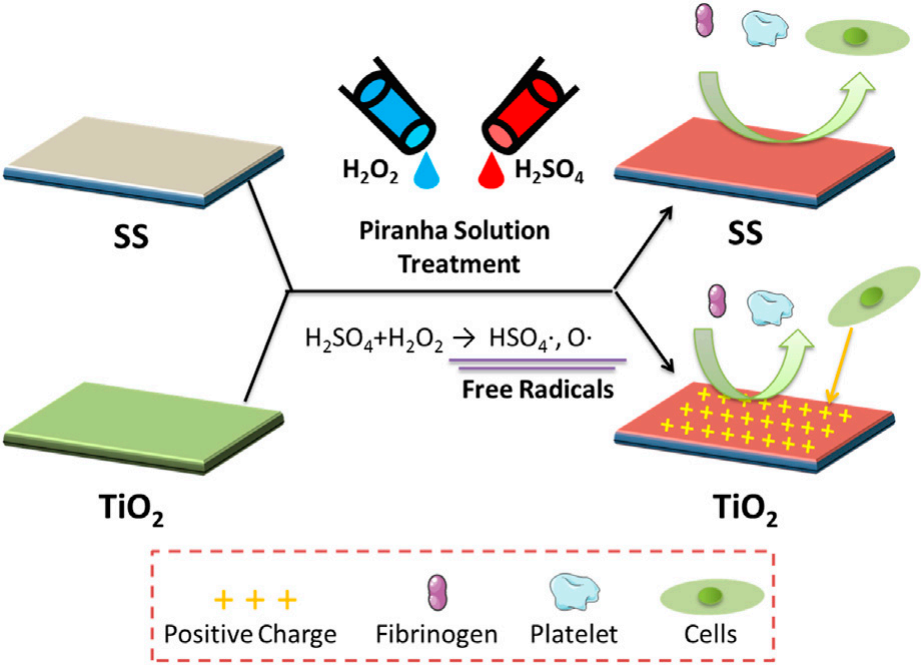
The possible mechanism underlying the attachment of the Fgn, platelet, and SMCs on the surface TiO_2_ and SS with piranha solution treatment.

The treatment of biomaterials with piranha solution was found to improve their antithrombotic properties, as evidenced by the results shown in Figure 5. Specifically, the treatment positively affected the blood compatibility of various inorganic materials but had little impact on organic materials like PTFE. Concerning cell affinity, the treatment had a different effect on different materials. The treatment of TiO_2_ with piranha solution (TiO_2_-piranha) was found to encourage the growth and proliferation of smooth muscle cells (SMCs), which was thought to be due to the positive charge property of the TiO_2_-piranha. While this enhancement in cell growth may not be desirable in using TiO_2_-piranha as a vascular stent, it suggests its potential use in fields where cell growth promotion is necessary, such as in developing bone implant materials. On the other hand, the treatment of SS with piranha solution (SS-piranha) effectively hindered the growth and proliferation of SMCs and was found to have strong antithrombotic properties. This makes SS-piranha a promising candidate for use as a blood implant device, such as a vascular stent.

## 4 Conclusion

The treatment with piranha solution was found to alter the surface hydrophilicity of inorganic materials efficiently and the oxidation state of organic adsorbates containing oxygen, thereby generally improving the antithrombotic properties of inorganic materials. Moreover, the treatment produced contrasting impacts on the cellular affinity of SS and TiO_2_. Specifically, it effectively decreased the adherence and proliferation of SMCs on SS surfaces. At the same time, it significantly increased the adherence and proliferation of SMCs on TiO_2_ surfaces, a phenomenon that may be attributed to the positive-charge property imparted to the TiO_2_ surface post-treatment. In conclusion, the treatment with piranha solution was demonstrated to be a straightforward, simple, and efficient means of enhancing the antithrombotic properties of inorganic materials while modulating their cytocompatibility. This holds promise for developing implantable medical devices that can fulfill the desired biological functions through judicious material selection.

## Data Availability

The raw data supporting the conclusion of this article will be made available by the authors, without undue reservation.

## References

[B1] Chen J. Yang P. Liao Y. Wang J. Chen H. Sun H. (2015). Effect of the duration of UV irradiation on the anticoagulant properties of titanium dioxide films. Acs Appl. Mater Inter 7 (7), 4423–4432. 10.1021/am509006y 25679095

[B2] Chen J. Zhao A. Chen H. Liao Y. Yang P. Sun H. (2014). The effect of full/partial UV-irradiation of TiO2 films on altering the behavior of fibrinogen and platelets. Colloids Surf. B. Biointerfaces 122 (0), 709–718. 10.1016/j.colsurfb.2014.08.004 25172575

[B3] Chen S. Sheng B. Qiu K. Liu Z. Xu X. Liu Y. (2011). Cleaning multilayer dielectric pulse compressor gratings with top layer of HfO 2 by Piranha solution. High Power Laser Part. Beams 23 (8), 2106–2110. 10.3788/hplpb20112308.2106

[B4] Cruz N. Gil J. Punset M. Manero J. M. Tondela J. P. Verdeguer P. (2022). Relevant aspects of piranha passivation in Ti6Al4V alloy dental meshes. Coatings 12 (2), 154. 10.3390/coatings12020154

[B5] Cui J. He S. Dai S. Liu L. Zhao A. Lu L. (2021). Stepwise assembly of functional proteins on Photo-activated TiO2 surfaces confers anti-oxidative stress ability and stealth effect to vascular stents. Chem. Eng. J. 424, 130392. 10.1016/j.cej.2021.130392

[B6] He Z. Lan X. Hu Q. Li H. Li L. Mao J. (2021). Antifouling strategies based on super-phobic polymer materials. Prog. Org. Coat. 157, 106285. 10.1016/j.porgcoat.2021.106285

[B7] Hori N. Ueno T. Minamikawa H. Iwasa F. Yoshino F. Kimoto K. (2010). Electrostatic control of protein adsorption on UV-photofunctionalized titanium. Acta Biomater. 6 (10), 4175–4180. 10.1016/j.actbio.2010.05.006 20466081

[B8] Iwasa F. Hori N. Ueno T. Minamikawa H. Yamada M. Ogawa T. (2010). Enhancement of osteoblast adhesion to UV-photofunctionalized titanium via an electrostatic mechanism. Biomaterials 31 (10), 2717–2727. 10.1016/j.biomaterials.2009.12.024 20035996

[B9] Kim S. Ye S.-h. Adamo A. Orizondo R. A. Jo J. Cho S. K. (2020). A biostable, anti-fouling zwitterionic polyurethane-urea based on PDMS for use in blood-contacting medical devices. J. Mater Chem. B 8 (36), 8305–8314. 10.1039/d0tb01220c 32785384 PMC7530005

[B10] Koh K.-S. Chin J. Chia J. Chiang C.-L. (2012). Quantitative studies on PDMS-PDMS interface bonding with piranha solution and its swelling effect. Micromachines-Basel 3 (2), 427–441. 10.3390/mi3020427

[B11] Liao Y. Li L. Chen J. Yang P. Zhao A. Sun H. (2017). Tailoring of TiO2 films by H2SO4 treatment and UV irradiation to improve anticoagulant ability and endothelial cell compatibility. Colloids Surf. B. Biointerfaces 155, 314–322. 10.1016/j.colsurfb.2017.04.021 28448901

[B12] Mou X. Zhang H. Qiu H. Zhang W. Wang Y. Xiong K. (2022). Mussel-inspired and bioclickable peptide engineered surface to combat thrombosis and infection. Research 2022, 9780879. 10.34133/2022/9780879 35515702 PMC9034468

[B13] Qiu H. Qi P. Liu J. Yang Y. Tan X. Xiao Y. (2019). Biomimetic engineering endothelium-like coating on cardiovascular stent through heparin and nitric oxide-generating compound synergistic modification strategy. Biomaterials 207, 10–22. 10.1016/j.biomaterials.2019.03.033 30947118

[B14] Qiu H. Tu Q. Gao P. Li X. Maitz M. F. Xiong K. (2021). Phenolic-amine chemistry mediated synergistic modification with polyphenols and thrombin inhibitor for combating the thrombosis and inflammation of cardiovascular stents. Biomaterials 269, 120626. 10.1016/j.biomaterials.2020.120626 33418199

[B15] Ross C. B. Sun L. Crooks R. M. (1993). Scanning probe lithography. 1. Scanning tunneling microscope induced lithography of self-assembled n-alkanethiol monolayer resists. Langmuir 9 (3), 632–636. 10.1021/la00027a002

[B16] Savaram K. Kalyanikar M. Patel M. Brukh R. Flach C. R. Huang R. (2015). Synergy of oxygen and a piranha solution for eco-friendly production of highly conductive graphene dispersions. Green Chem. 17 (2), 869–881. 10.1039/c4gc01752h

[B17] Yang Y. Gao P. Wang J. Tu Q. Bai L. Xiong K. (2020). Endothelium-mimicking multifunctional coating modified cardiovascular stents via a stepwise metal-catechol-(amine) surface engineering strategy. Research 2020, 9203906. 10.34133/2020/9203906 32405627 PMC7196174

[B18] Yang Z. Yang Y. Xiong K. Wang J. Lee H. Huang N. (2018). Metal-phenolic surfaces for generating therapeutic nitric oxide gas. Chem. Mater. 30 (15), 5220–5226. 10.1021/acs.chemmater.8b01876

